# VS-5584, a Novel PI3K-mTOR Dual Inhibitor, Inhibits Melanoma Cell Growth *In Vitro* and *In Vivo*

**DOI:** 10.1371/journal.pone.0132655

**Published:** 2015-07-23

**Authors:** Zheren Shao, Qi Bao, Fangzhen Jiang, Huan Qian, Quan Fang, Xueqing Hu

**Affiliations:** Department of Plastic Surgery, The Second Affiliated Hospital, Medical School, Zhejiang University, Hangzhou, China; Suzhou University, CHINA

## Abstract

Melanomas cause over 76% of skin cancer deaths annually. Phosphatidylinositol 3-kinase (PI3K)-AKT-mammalian target of rapamycin (mTOR) signaling pathway is important for melanoma initiation and progression. In the current study, we evaluated the potential anti-melanoma effect of VS-5584, a novel and highly potent PI3K-mTOR dual inhibitor. We demonstrated that VS-5584 potently inhibited survival and proliferation of established (A375, A-2058 and SK-MEL-3 lines) and primary human melanoma cells, but was non-cytotoxic to non-cancerous human skin keratinocytes and B10BR murine melanocytes. At the meantime, VS-5584 induced caspase-dependent apoptotic death in melanoma cells, and its cytotoxicity was alleviated by the caspase inhibitors. At the molecular level, VS-5584 blocked AKT-mTOR activation and downregulated cyclin D1 expression in melanoma cells, while the expressions of Bcl-xL and Bcl-2 were not affected by VS-5584 treatment. On the other hand, a BH-3 mimetic Bcl-xL/Bcl-2 inhibitor ABT-737, as well as siRNA-mediated knockdown of Bcl-xL or Bcl-2, enhanced the activity of VS-5584 in melanoma cells. *In vivo*, oral administration of VS-5584 suppressed A375 melanoma xenograft growth in nude mice, and its activity was further enhanced by co-administration of ABT-737. These results provide the rationale for the clinical assessment of VS-5584 in melanoma patients and development of ABT-737 and other Bcl-xL/Bcl-2 inhibitors as the possible adjuvants.

## Introduction

Only less than 5% of skin cancers are melanomas, however, the latter cause over 76% of skin cancer deaths annually [1]. Meanwhile, the incidence of melanoma continues to rise at an alarming rate [1]. It is estimated that more than 75,000 new melanomas will be diagnosed in the year 2015 in United States alone, and around 10,000 people are expected to die because of this devastating disease [1]. Further, melanoma is among the most resistance cancers to possible all-known chemotherapeutic agents [2,3,4,5]. Thus, there is an urgent need to explore other alternative and targeted therapies [2,3,4,5].

The phosphoinositide 3-kinase (PI3K)-AKT signaling plays a vital role in regulating many aspects of cancer behaviors, including cell growth, survival and chemoresistance [6,7,8,9]. It is also vital to diverse physiologic processes including cell-cycle progression, differentiation, transcription and translation [6,7,8,9]. Dysregulation of this pathway has been closely associated with initiation and progression of melanoma [9,10] and other cancers [6,7,11]. A number of genetic alterations in players of this signaling pathway have been identified, including p85, p110α, PDK1, PTEN, and AKT [6,7,11]. The dysregulated PI3K-AKT pathway causes aberrant activation of downstream effectors, including the most-extensively studied one mammalian target of rapamycin (mTOR) [12]. The availability of many inhibitors against this pathway has led to the evaluation of their activity against melanoma in pre-clinical settings and clinical trials [8].

VS-5584 is a novel low-molecular weight compound, which shows extremely potent, highly-selective inhibition against both PI3K and mTOR [13]. Unlike the currently available clinical stage compounds, it displays almost equivalent activity against PI3K and mTOR [13]. Sporadic studies have shown that it has very good pharmacokinetic properties, and is well-tolerated in animal models [13]. In the current study, we investigated the potential activity of this PI3K-mTOR dual inhibitor against melanoma cells *in vitro* and *in vivo*.

## Materials and Methods

### 2.1. Chemicals and reagents

VS-5584 and ABT-737 were obtained from Selleck (Shanghai, China). Both compounds were dissolved in DMSO and stored at -20° for *in vitro* studies. For *in vivo* studies, both agents were dissolved in the SX-1292 oral vehicle [1% sodium carboxymethyl cellulose, 0.5% sodium lauryl sulfate (SLS), and 0.05% antifoam; Eli Lilly, Shanghai, China] and administered by gastric lavage [13,14]. Antibodies of p-AKT (Ser 473, #9271), p-AKT (Thr 308, #9275), p-S6 ribosomal protein (S6, Ser 235/236, #2211), S6 (#2317), p-p70 S6 kinase 1 (S6K1, Thr 398, #9209), S6K1 (#2708), Bcl-2 (#2870), Bcl-xL (#2762) and glyceraldehyde-3-phosphate dehydrogenase (GAPDH, #2118) were obtained from Cell Signaling Technology (Danvers, MA). The concentration of primary antibodies utilized in this study was 1: 1,000, except for p-S6 (1: 10,000) and GAPDH (1: 10,000). The caspase inhibitors z-VAD-fmk and z-DVED-fmk were obtained from Calbiochem (Shanghai, China).

### 2.2. Cell culture

The melanoma cell lines A375, A-2058 and SK-MEL-3, as well as B10BR mouse melanocytes and primary human keratinocytes were purchased from ATCC. Melanoma cells were cultured in DMEM/RPMI supplemented with 10% fetal bovine serum (FBS, Gibco-BRL, Shanghai, China), penicillin-streptomycin (100 U/mL penicillin and 100 μg/mL streptomycin) and 2 mM L-glutamine, and grown in a humidified atmosphere of air containing 5% CO_2_ at 37°C. Melanoma cells were stained with trypan blue, and viable cells were trypan blue exclusive.

B10BR melanocytes were cultured in Hams F12 supplement with 7% heat-inactivated calf serum (FCS), 50 ng/mL phorbol 12-myristate 13-acetate (TPA) and 1% penicillin/streptomycin.

Primary human keratinocytes were maintained in Medium 154-CF (Cascade Biologics, Portland, OR) supplemented with Human Keratinocyte Growth Supplement (HKGS, Cascade Biologics) plus antibiotics and Ca^2+^ (0.03 mM).

The primary human melanoma cells were isolated after surgery from subcutaneous metastasis of three independent patients with uveal melanoma (Patient-1: male, 38 years old; Patient-2, male, 45 years old; and Patient-3, female, 40 years old). After collagenase digestion, primary tumor cells were maintained in F12 medium containing 20% FBS. After 5 days in culture, non-adherent cells were removed, retaining the adherent fraction for further study (passage number < 4). Experiments and the protocols requiring clinical samples were approved by the ethics committee of Medical School, Zhejiang University (Ling Xu, Bing Wang, and Wei Liu). Each participant provided written informed consent to participate the current study, which was approved the ethics committee.

### 2.3. Cell survival assay

Cell survival was determined by 3-(4,5-dimethylthiazol-2-yl)-2, 5-diphenyltetrazolium bromide (MTT, Sigma Chemical Co., St. Louis, MO) assay. Briefly, cells were placed onto a 96-well plate at 5 × 10^3^ cells per well. After the treatment, cells were incubated for 90 min with MTT reagent (300 μL, 0.5 mg/mL). The MTT solution was removed and the formazan crystals were dissolved in DMSO, and absorbance was recorded at 570 nm on a Dynatech mini-microplate reader. The OD value of treatment group was normalized to that of the vehicle control group.

### 2.4. [H^3^] Thymidine incorporation assay

Cells were incubated with indicated treatment plus 1 μCi/mL of tritiated thymidine. After the treatment, cells were washed with cold PBS for three times, the DNA was precipitated with cold 10% trichloroacetic acid (TCA), solubilized with 1.0 M sodium hydroxide, and aliquots were counted by liquid-scintillation spectrometry. The value of treatment group was normalized to that of vehicle control group.

### 2.5. Clonogencity assay

Cells were plated in 6-well plates at 1000 cells per well, which were then treated with gradient concentrations of VS-5584. Eight days after the treatment, survival colonies were fixed with 3% glutaraldehyde, 0.2% crystal violet and 20% methanol, and were manually counted.

### 2.6. Annexin V-FITC flow cytometry analysis of cell apoptosis

Following the treatment, cells (2×10^5^/sample) were harvested and washed twice with ice-cold PBS. The Annexin V-FITC apoptosis detection kit (Molecular Probes, Eugene, OR) was utilized for detecting apoptotic cells. Briefly, 20 μL aliquots of Annexin V-FITC and 40 of μL propidium iodine (PI) buffer were added to 400 μL of binding buffer, which was then added to the cells for 15 min in the dark at room temperature. Samples were analyzed with fluorescence-activated cell sorting (FACS; Becton-Dickinson). Annexin V percentage was recorded as a measurement of cell apoptosis.

### 2.7 ELISA assay of apoptosis

Fragmented DNA was assessed by measuring DNA-associated with nucleosomal histones using a specific two-step enzyme-linked immunosorbent assay (ELISA) with an anti-histone primary antibody and a secondary anti-DNA antibody, according to the manufacturer's instructions (Roche Applied Science, Shanghai, China). ELISA OD at 450 nm was recorded as a quantitative measurement of apoptosis.

### 2.8. Caspases activity assay

Cellular caspase activity was measured by the chromophore-labeled specific substrates, including Asp-Glu-Val-Asp p-nitroanilide (DEVD-pNA, the substrate for caspase-3), and Leu-Glu-His-Asp p-nitroanilide (LEHD-pNA, the substrate for caspase-9). After the treatment, cells were incubated with a 200-μL lysis buffer at 4°C for 20 min. The cellular proteins were then analyzed with the chromophore-labeled specific substrates according to the manufacturer’s protocols (BioVision, Inc., Milpitas, CA). The spectrophotometric analysis was performed at 405 nm, and the caspase activity was expressed as the fold change of the vehicle control.

### 2.9. Protein extraction and Western blots

The treated cells or tumor tissue samples were homogenized on ice in ice-cold lysis buffer (50 mM Tris–HCl, 150 mM NaCl, 1 mM EGTA, 1 mM EDTA, 20 mM NaF, 100 mM Na3VO4, 0.5% NP-40, 1% Triton X-100, 1 mM PMSF, pH 7.4) with freshly-added protease inhibitor cocktail (Roche, Shanghai, China). For Western blot, 30 μg protein per sample was resolved over 10% SDS-page gels and transferred to a PVDF membrane. The blot was blocked in blocking buffer (10% non-fat dry milk/1% PBST) for 1 hour, incubated with appropriate primary antibody overnight at 4°C, followed by incubation with horseradish peroxidase-conjugated secondary antibody. The Western blot signal was detected by enhanced Super-signal enhanced chemiluminescence (ECL) reagent. The intensity of each band was quantified through ImageJ software (NIH) after normalized to the corresponding loading control.

### 2.10. RNA interference (RNAi)

A375 cells were cultured onto a 6-well plate, and transfected with targeted small-interfering RNA (siRNA) oligonucleotides for Bcl-2 (SignalSilence Bcl-2 siRNA I, Cell Signaling-6441), Bcl-xL (SignalSilence Bcl-xL siRNA II, Cell Signaling-6363) or scramble control siRNA (sc-RNAi, Santa Cruz Biotech, Santa Cruz, CA) using Lipofectamine 2000 (Invitrogen, USA) for 48 hours according to the manufacturer's instructions. The efficiency of the siRNA was tested by Western blots.

### 2.11. Tumor xenograft study

Male nude mice were housed under pathogen-free conditions with a 12-hour light/dark cycle. A total of 2 × 10^6^ of A375 cells (in 100 μL DMEM + 100 μL Matrigel) were implanted subcutaneously into the right flanks. Mice bearing A375cells were randomly divided into four groups of ten mice per group. 14 days post inoculation when tumor volumes reached around 200 mm^3^, animals of group I received SX-1292 and served as vehicle control, group II received ABT-737 (25 mg/kg, lavage daily) [15], group III received VS-5584 (25 mg/kg, lavage daily) [13], and group IV received ABT-737 plus VS-5584. Treatments were continued for a total of three weeks. The tumor volume was calculated using the formula: V = 0.5328 × Long × Width × High (mm^3^). The animals were also evaluated for body weights, consumption of food to access apparent signs of toxicity. This study was approved by the regulation of the Institutional Animal Care and Use Committee (IACUC) of Medical School, Zhejiang University (Contact person: Li Li).

### 2.12 Statistical analysis

The data presented were mean ±standard deviation (SD). Statistical differences were analyzed by one-way ANOVA followed by multiple comparisons performed with post hoc Bonferroni test (SPSS version 18.0, Chicago, CA). Values of *p*<0.05 were considered statistically significant. The significance of any differences between two groups was tested using paired-samples t test when appropriated.

## Results

### 3.1. VS-5584 inhibits melanoma cell survival and proliferation

**Fig 1:**
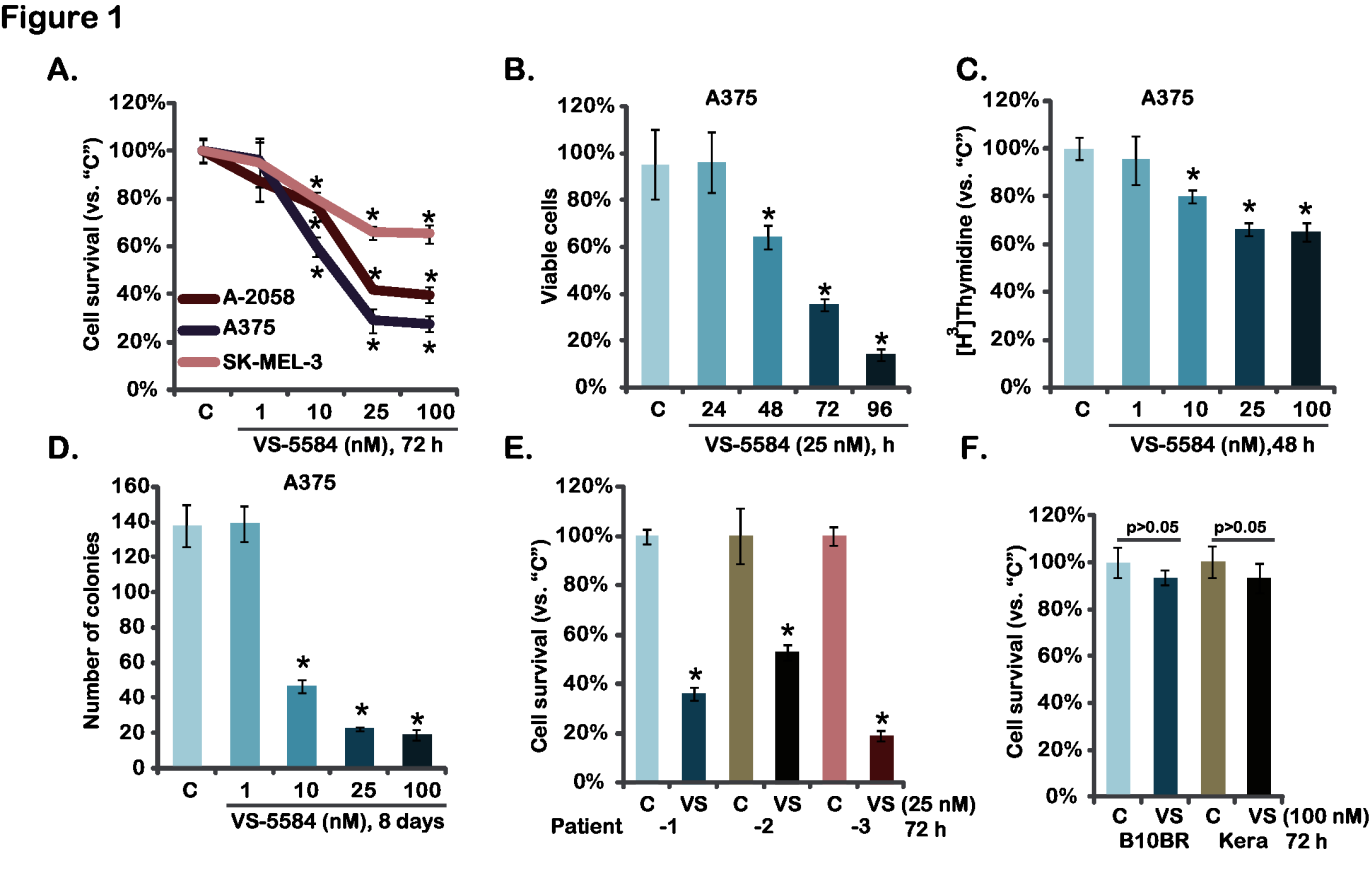
VS-5584 inhibits melanoma cell survival and proliferation-Established melanoma cell lines (A375, A-2058 and SK-MEL-3), patient-derived primary melanoma cells, B10BR melanocytes and primary human keratinocytes (“Kera”) were treated with applied concentration of VS-5585 (“VS”) or vehicle control (“C”, 0.1% of DMSO), cell survival was tested by MTT assay (A, E and F) and trypan blue exclusion assay (B, for A375 cells); Cell proliferation was analyzed by through [H^3^] Thymidine incorporation assay (C, for A375 cells) and clonogenicity assay (D, for A375 cells). Data were expressed as mean ± SD, experiments were repeated three times. *p<0.05 vs group “C”.

As discussed early, activation of PI3K-AKT-mTOR pathway is a major contributor for melanoma progression [9,16]. VS-5584 is newly developed PI3K-mTOR dual inhibitor [17]. We first examined the potential effect of VS-5584 on melanoma cell survival, which was tested by measuring levels of MTT incorporation into the cells. As shown in Fig 1A, VS-5584 potently inhibited survival of three established melanoma cell lines (A375, A-2058 and SK-MEL-3). The effect of this PI3K-mTOR dual inhibitor was dose-dependent, and it was most potent in A375 cells (Fig 1A). Its activity was also time-dependent, and VS-5584 took around 48 hours to achieve anti-survival activity in A375 cells (Fig 1B). PI3K-AKT-mTOR plays a vital role in cancer cell proliferation [9,18]. Thus, the potential effect of VS-5584 on melanoma cell proliferation was also analyzed through [H^3^] Thymidine incorporation assay (Fig 1C) and clonogenicity assay (Fig 1D). Results from both assays showed that VS-5584 at the concentrations between 10–100 nM remarkably inhibited A375 melanoma cell proliferation (Fig 1C and 1D). Similar anti-proliferation results were also obtained in A-2058 and SK-MEL-3 melanoma cells (see clonogenicity assay results in S1 Fig). The potential activity of VS-5584 on patient-derived primary melanoma cells was also analyzed (Fig 1E). VS-5584 was cytotoxic to all three primary human melanoma cell lines (Fig 1E). Notably, VS-5584 at 100 nM was non-cytotoxic to two non-cancerous cell lines: primary human skin keratinocytes and B10BR murine melanocytes (Fig 1F). Collectively, we show that VS-5584 inhibits melanoma cell survival and proliferation.

### 3.2. VS-5584 induces caspase-dependent apoptotic death of melanoma cells

**Fig 2:**
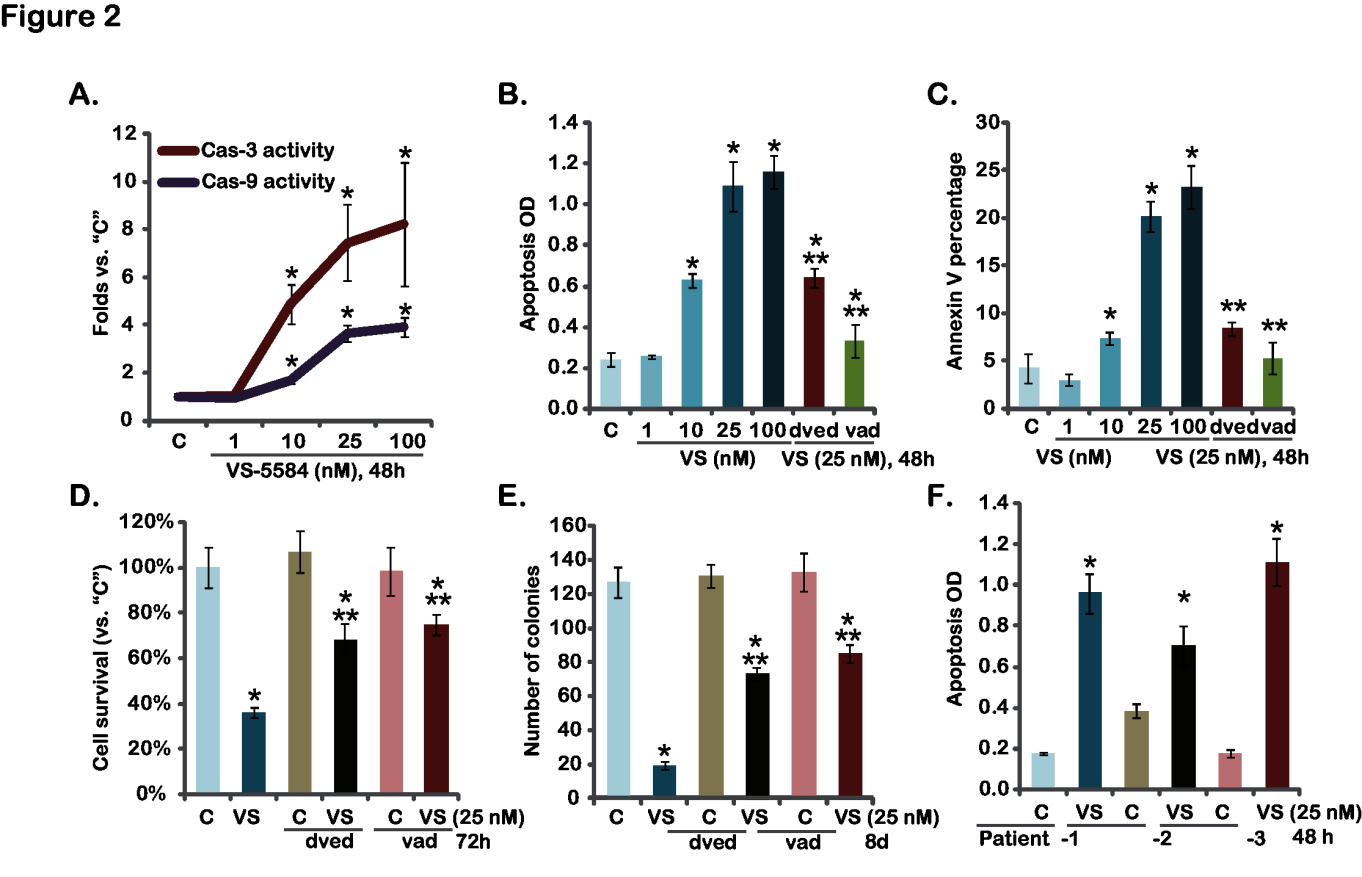
VS-5584 induces melanoma cell apoptosis-A375 cells, pre-treated with the caspase-3 inhibitor z-DVED-fmk (“dved”, 60 μM), the pan caspase inhibitor z-VAD-fmk (“vad”, 60 μM), were treated with applied concentration of VS-5584 (“VS”) for indicated time, caspase-3/-9 activity was analyzed as described (A), cell apoptosis was tested by Histone-DNA ELISA assay (B) and Annexin V FACS assay (C), cell survival and proliferation were tested by MTT assay (D) and clonogenicity assay (E), respectively. Patient-derived primary melanoma cells were treated with VS-5584 (“VS”, 25 nM) or vehicle control (“C”, 0.1% of DMSO) for 48 hours, cell apoptosis was assayed by Histone-DNA ELISA assay (F). Data were expressed as mean ± SD, experiments were repeated three times. *p<0.05 vs group “C”. **p<0.05 vs “VS” (25 nM) only group (B-E).

The effect of VS-5584 on melanoma cell apoptosis was also tested. Activation of caspases is an important feature of apoptosis. Thus, the activity of caspase-3 and caspase-9 in VS-5584-treated melanoma cells was tested. Results in Fig 2A demonstrated that VS-5584 dose-dependently increased activities of caspase-3/-9 in A375 cells. Meanwhile A375 cell apoptosis, tested by the Histone-DNA ELISA assay and Annexin V FACS assay, was induced by VS-5584 treatment (Fig 2B and 2C). Similar results were also observed in A-2058 and SK-MEL-3 melanoma cells (Data not shown). The specific caspase-3 inhibitor z-DVED-fmk (“dved”) and the pan caspase inhibitor z-VAD-fmk (“vad”) remarkably inhibited VS-5584-induced A375 cell apoptosis (Fig 2B and 2C). As a result, VS-5584-induced cytotoxicity, tested by the MTT assay (Fig 2D) and the clonogenicity assay (Fig 2E), was alleviated by the two caspase inhibitors (Fig 2D and 2E). VS-5584 also induced significant apoptosis in primary human melanoma cells (Fig 2F). Together, these results show that VS-5584 induces caspase-dependent apoptotic death of melanoma cells.

### 3.3. VS-5584 blocks AKT-mTOR activation in melanoma cells

**Fig 3:**
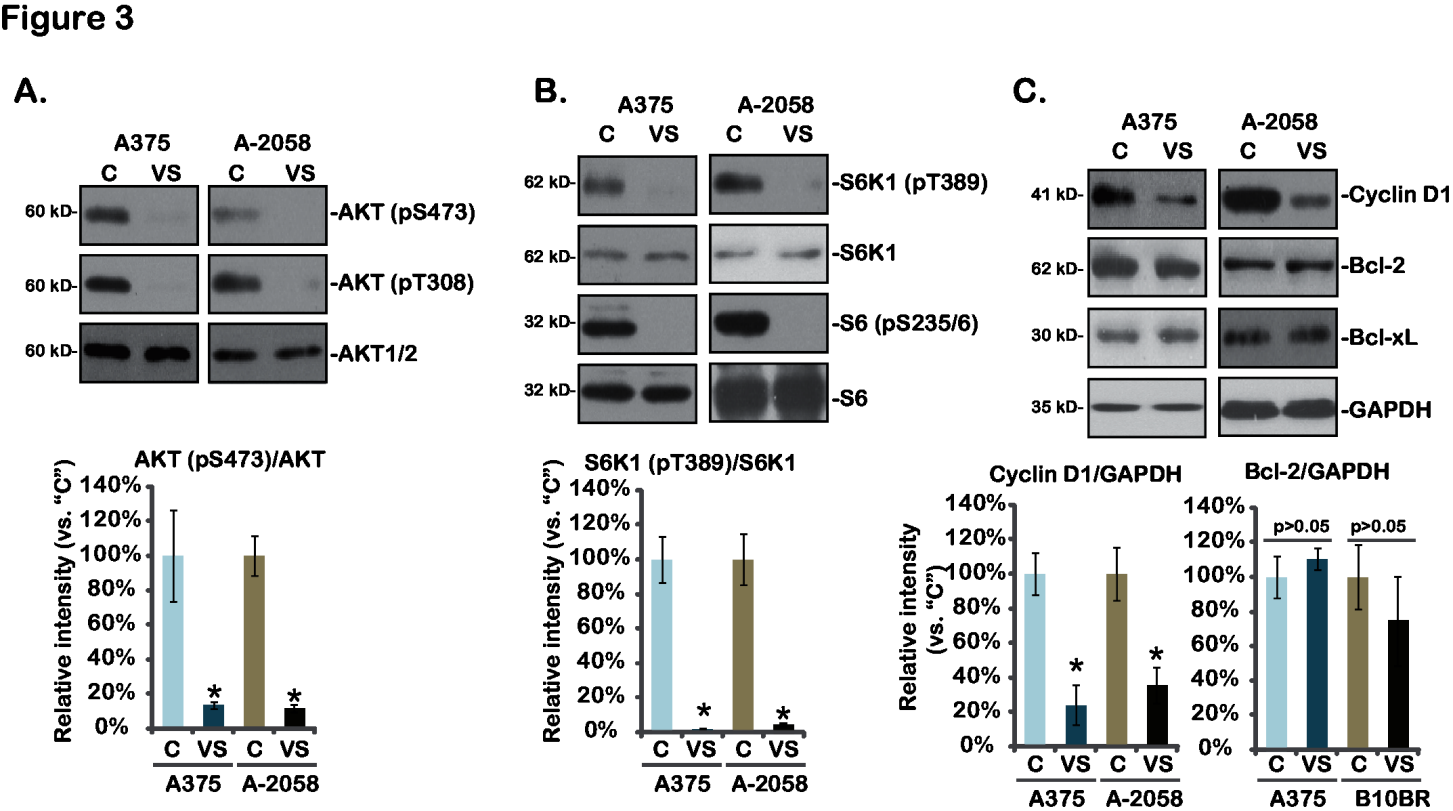
Signaling changes by VS-5584 in melanoma cells-A375 and A-2058 cells were treated with VS-5584 (“VS”, 25 nM) or the vehicle control (1% DMSO, “C”) for 6 hours (for A and B) or 24 hours (for C), expression of listed proteins was tested by Western blots. Relative intensity of kinase phosphorylations (vs. non-phosphorylated kinases) as well as cyclin D1 and Bcl-2 expression (vs. GAPDH) of three independent experiments were shown (A-C, lower panels). Data were expressed as mean ± SD, experiments were repeated three times. *p<0.05 vs group “C”.

VS-5584 is a novel PI3K-mTOR dual inhibitor [13,17]. Next, AKT-mTOR activation in VS-5584-treated melanoma cells was analyzed. As shown in Fig 3A, AKT activation, indicated by phosphorylation at both Ser-473 and Thr-308, was almost completely blocked by VS-5584 in A375 and A-2058 melanoma cells (Fig 3A). Further, phosphorylations of S6K1 and S6, both indicators of mTOR complex 1 (mTORC1) activation (see below), were dramatically inhibited by VS-5584 in melanoma cells (Fig 3B). At the meantime, cyclin D1, a mTORC1-regulated gene [19], was downregulated by VS-5584 in the two melanoma cell lines. On the other hand, expressions of anti-apoptosis proteins including Bcl-2 and Bcl-xL were not affected by same VS-5584 treatment (Fig 3C). These results together show that VS-5584 blocks AKT-mTOR activation and down-regulates cyclin D1 expression in melanoma cells.

### 3.4. Bcl-xL/Bcl-2 inhibition sensitizes VS-5584-mediated activity in melanoma cells

**Fig 4:**
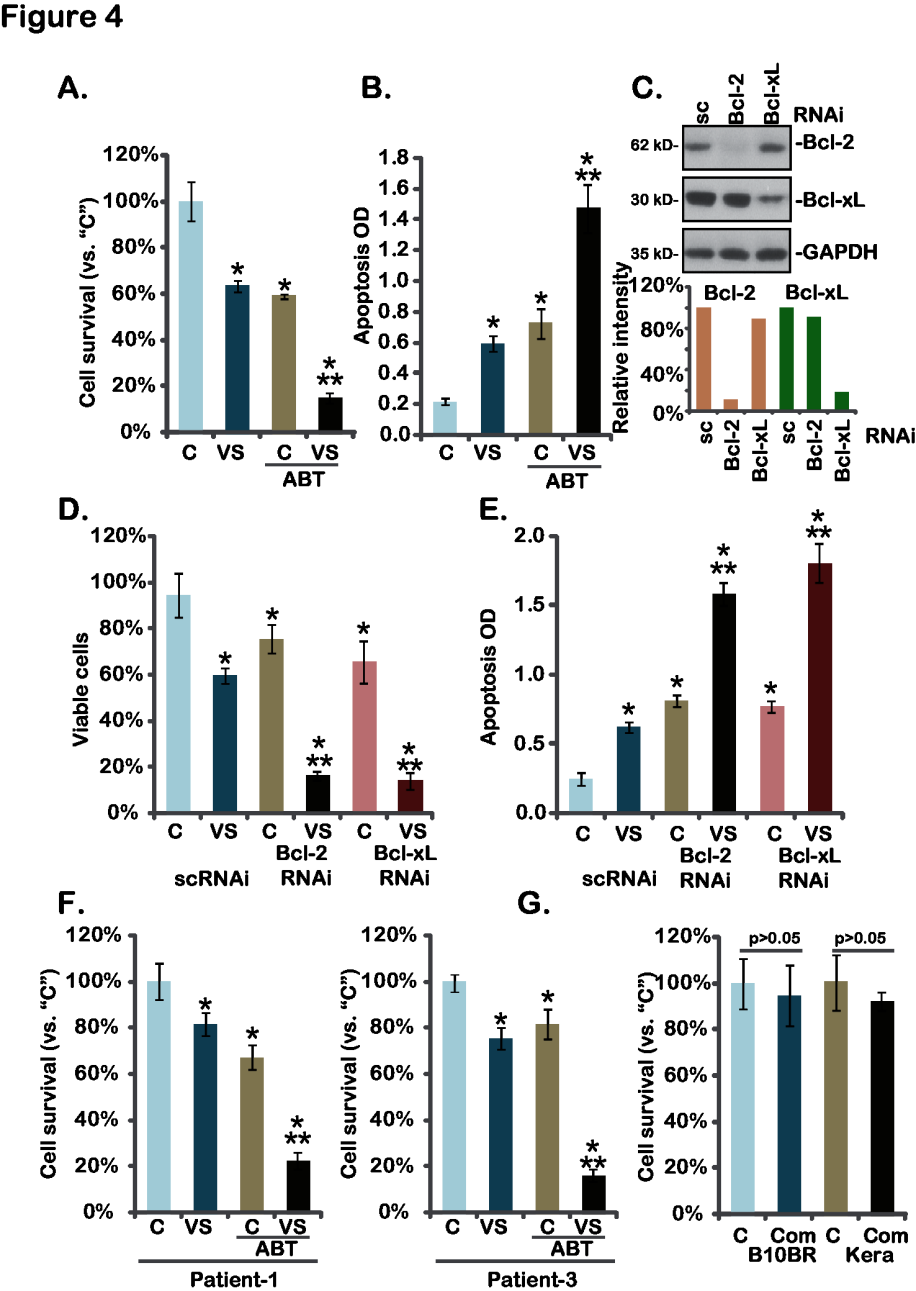
Bcl-xL/Bcl-2 inhibition sensitizes VS-5584-mediated activity in melanoma cells-A375 cells (A and B), primary human melanoma cells (F), B10BR murine melanocytes (G) or primary human keratinocytes (“Kera”) (G) were treated with VS-5584 (“VS”, 10 nM) or plus ABT-737 (“ABT”, 25 nM) for 72 hours, cell viability (MTT assay) and apoptosis (ELISA assay, for A375 cells) were tested. A375 cells, transfected with scramble control siRNA (sc-RNAi), Bcl-2 siRNA or Bcl-xL siRNA, were treated with VS-5584 (VS, 10 nM) for 72 hours, expression of Bcl-2, Bcl-xL and GAPDH was tested by Western blots (C, their expressions were quantified), cell survival (D) and apoptosis (E) were also tested. Data were expressed as mean ± SD, experiments were repeated three times. “C” stands for vehicle control (0.1% of DMSO). *p<0.05 vs group “C”. **p<0.05 vs “VS” only group (A, B and F). **p<0.05 vs sc-RNAi group (D and E).

Above results have shown that expressions of Bcl-xL and Bcl-2 were not affected by the VS-5584 treatment in melanoma cells. Bcl-xL and Bcl-2 play an important role in melanoma cell resistance [20]. Thus, we tested whether Bcl-xL/Bcl-2 inhibition could further increase the activity of VS-5584. ABT-737, a BH-3 mimetic inhibitor of Bcl-xL and Bcl-2 [21], was applied. Results showed that treatment with ABT-737 (100 nM) alone induced moderate death and apoptosis in A375 cells (Fig 4A and 4B). Significantly, same ABT-737 treatment dramatically enhanced VS-5584 (10 nM)-induced activity against A375 cells, leading to substantial cell death and apoptosis (Fig 4A and 4B). Similar VS-5584-senstization activity by ABT-737 was also seen in A-2058 and SK-MEL-3 melanoma cells (Data not shown). These results suggest that Bcl-xL/Bcl-2 inhibition by ABT-737 could sensitize the anti-melanoma activity of VS-5584. To further support our hypothesis, siRNA method was applied. Results demonstrated that siRNA-mediated knockdown of Bcl-2 or Bcl-xL (Fig 4C) remarkably enhanced VS-5584-induced viability reduction (Fig 4D) and apoptosis (Fig 4E) in A375 cells, further confirming that Bcl-xL/Bcl-2 silence leads to VS-5584 sensitization. In primary human melanoma cells, ABT-737 and VS-5584 synergistically inhibited melanoma cell survival (Fig 4F). While in primary human keratinocytes and B10BR melanocytes, same VS-5584 and ABT-737 combination (“Com”) showed no activity on cell survival (Fig 4G). These results demonstrate that Bcl-xL/Bcl-2 inhibition sensitizes VS-5584-mediated activity in melanoma cells.

### 3.5. VS-5584 inhibits A375 xenograft growth in nude mice, and its activity was further enhanced by co-administration of ABT-737

**Fig 5:**
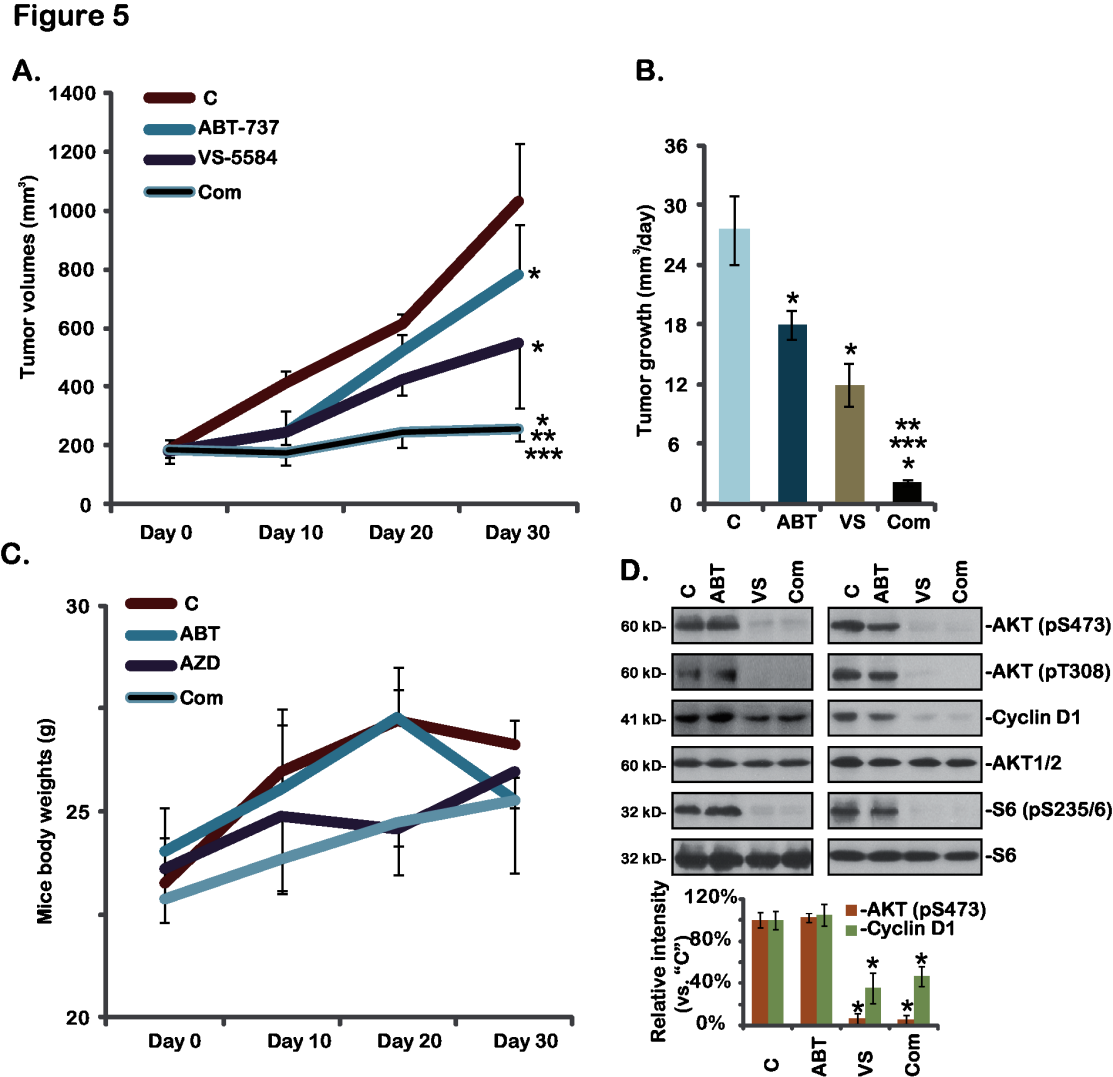
VS-5584 and ABT-737 synergistically inhibits A375 xenograft growth *in vivo*-A375 bearing nude mice (n = 10 for each group) were treated with vehicle control (SX-1292, “C”), VS-5584 (“VS”, oral, 25 mg/kg/d for 21 days), ABT-737 (“ABT”, oral, 25 mg/kg/d for 21 days), or VS-5584 plus ABT-737 combo (“Com”), tumor volumes (A and B) and mice body weights (C) were recorded. Two weeks after initial drug administration, xenografted tumors of two mice per group were isolated, expressions of listed proteins in the tumor lysates were tested and quantified (D). Data were expressed as mean ± SD, experiments were repeated twice. *p<0.05 vs group “C”. **p<0.05 vs “VS-5584” only group. ***p<0.05 vs “ABT-737” only group.

At last, we tested the *in vivo* activity of VS-5584 using an A375 xenograft mouse model (See material and method). The results in Fig 5A demonstrated that oral administration of a single dose of VS-5584 (25 mg/kg) inhibited A375 xenograft growth in nude mice. Daily tumor growth volume was also decreased when treatment with VS-5584 in mice (Fig 5B). Remarkably, co-administration of ABT-737 further enhanced the *in vivo* activity of VS-5584, resulting in dramatic inhibition of A375 xenograft growth (Fig 5A and 5B). The combined activity was more potent than either agent alone (Fig 5A and 5B). ABT-737 (25 mg/kg) alone only showed moderate activity against A375 xenografts (Fig 5A and 5B). Notably, mice body weights were not affected by the single or combined treatment, indicating the relative safety of the regimens (Fig 5C). Western blots assaying xenografted tumor lysates showed that AKT-S6 activations as well as cyclin D1 expression were significantly inhibited by VS-5584 or plus ABT-737 treatment *in vivo* (Fig 5D), which are consistent with the *in vitro* findings. Thus, VS-5584 oral administration inhibits A375 xenograft growth *in vivo*, its activity could be further boosted by co-administration of ABT-737.

## Discussions

Malignant melanoma is highly resistant to current chemotherapeutic agents [2,3,4,5]. Dysregulation of the PI3K-AKT-mTOR pathway, either through amplifications (i.e. *ERBB2*), deletions (i.e. *PTEN*), or as a direct result of mutations (i.e. *PI3KCA*), has been closely linked to the development, progression and chemoresistance of melanoma [9,22]. Moreover, over activation of this pathway is often a poor prognostic marker for melanoma [9,22]. In the current study, we showed that VS-5584, a novel PI3K-mTOR dual inhibitor, significantly inhibited PI3K-AKT-mTOR activation and melanoma cell growth *in vitro* and *in vivo*.

mTOR is in the two distinct complexes, namely mTOR complex 1 (mTORC1) and mTORC2 [23,24]. mTORC1, a rapamycin-sensitive complex that is composed of mTOR, raptor and mLST8 and others, regulates cellular growth by integrating signals from growth factor receptors and intracellular nutrients [23,24]. mTORC2 comprises mTOR, Rictor, Sin1 and others, serving as the upstream kinase of AKT at Ser 473, and is important in regulating cellular survival, growth and migration [6,25,26]. Inhibitors of mTOR pathway have been developed to target melanoma and other cancers [22]. The first generation of mTOR inhibitors, including rapamycin and its analogs, block mTORC1 activity, but only show moderate activity in a small subsets of cancers [12,26]. Resistance has been developed through feedback activation of the PI3K, mTORC2 as well as Erk-MAPK signaling pathways due to mTORC1 inhibition [12,26]. In the current study, we showed that VS-5584 treatment almost completely blocked activation of mTORC1, indicated by p-S6K1 and p-S6, and mTORC2 or p-AKT-Ser473 in melanoma cells. Further, VS-5584 simantanuously inhibited p-AKT T-308 activation. These could explain, at least in part, the potent activity of this PI3K-mTOR dual inhibitor in melanoma cells.

Overexpression of anti-apoptotic proteins including Bcl-xL and Bcl-2 was observed in many melanomas, which correlates to cancer progression [20]. Bcl-xL and Bcl-2 are both important for tumor progression, and chemoresistance through interacting with pro-apoptotic BH3 proteins [20]. In the current study, we showed that Bcl-xL/Bcl-2 could be one major resistance factor of VS-5584. VS-5584 failed to affected Bcl-xL and Bcl-2 expressions in tested melanoma cells. Importantly, the Bcl-xL/Bcl-2 inhibitor ABT-737, or siRNA-mediated knockdown of Bcl-xL/Bcl-2, remarkably enhanced the activity of VS-5584 against melanoma cells *in vitro* and *in vivo*. Collectively, these results indicate that inhibition of Bcl-xL/Bcl-2 could significantly sensitize VS-5584’s activity against melanoma cells. Further studies will be needed to test the underlying mechanisms of VS-5584 resistance by Bcl-xL/Bcl-2, and to repeat these results in other cancer cells.

## Conclusions

In summary, the results of this study demonstrate that VS-5584 inhibits melanoma cell proliferation probably through blocking PI3K-AKT-mTOR signaling pathway. Inhibition of Bcl-2/Bcl-xL could further enhance the activity of VS-5584 against melanoma cells *in vitro* and *in vivo*. Our data suggest that VS-5584, or plus Bcl-2/Bcl-xL inhibitors, may be beneficial for patients with melanoma.

## Supporting Information

S1 Fig SK-MEL-3 and A-2058 melanoma cells were treated with applied concentration of VS-5585 or vehicle control (“C”, 0.1% of DMSO), cell proliferation was analyzed by the clonogenicity assay. Data were expressed as mean ± SD, experiments were repeated three times. *p<0.05 vs group “C”.
(EPS)
